# Implementation of an Automated Dispensing Cabinet System and Its Impact on Drug Administration: Longitudinal Study

**DOI:** 10.2196/24542

**Published:** 2021-09-17

**Authors:** Yi-Chen Wang, Chin-Yuan Tsan, Meng-Chun Chen

**Affiliations:** 1 Department of Nursing National Taiwan University Hospital Taipei City Taiwan; 2 Department of Nursing National Taiwan University Hospital, Yunlin Branch Yunlin County Taiwan

**Keywords:** automated dispensing cabinets, medication administration system, medication errors, dispensing, medication, nursing, Taiwan

## Abstract

**Background:**

A technology that has been widely implemented in hospitals in the United States is the automated dispensing cabinet (ADC), which has been shown to reduce nurse drug administration errors and the time nurses spend administering drugs.

**Objective:**

This study aimed to determine the impact of an ADC system on medication administration by nurses as well as safety before and after ADC implementation.

**Methods:**

We conducted a 24-month-long longitudinal study at the National Taiwan University Hospital in Taipei, Taiwan. Clinical observations and questionnaires were used to evaluate the time differences in drug preparation, delivery, and returns in the inpatient ward by nurses before and after using the ADC. Drug errors recorded in the Medical Incident Events system were assessed the year before and after ADC implementation.

**Results:**

The drug preparation time of the wards increased significantly (all *P*<.005). On average, 2 minutes of preparation time is needed for each patient. Only 1 unit showed an increase in the drug return time, but this was not significant. There were 9 (45%) adverse events during the drug administration phase, and 11 (55%) events occurred during the drug-dispensing phase. Although a decrease in the mean number of events reported was observed during the ADC implementation period, this difference was not significant. As for the questionnaire that were administered to the nurses, the overall mean score was 3.90; the highest score was for the item “I now spend less time waiting for medications that come from the pharmacy than before the ADC was implemented” (score=4.24). The item with the lowest score was “I have to wait in line to get my patient medications” (score=3.32).

**Conclusions:**

The nurses were generally satisfied with ADC use over the 9 months following complete implementation and integration of the system. It was acknowledged that the ADC offers benefits in terms of pharmaceutical stock management; however, this comes at the cost of increased nursing time. In general, the nurses remained supportive of the benefits for their patients, despite consequences to their workflows. Their acceptance of the ADC system in this study demonstrates this.

## Introduction

Although the Joint Commission on Accreditation of Healthcare Organizations promotes medication administration safety as one of its key standards to improve patient safety, there are still 400,000 drug-related adverse events in the United States yearly, with annual costs estimated at US $3.5 billion. According to an investigation by Taiwan’s Ministry of Health and Welfare, from 2014 to 2019, there were more than 20,000 adverse drug events (ADEs) every year. Some studies found that many serious medical errors—which cause or have the potential to cause damage or injury—are drug prescription or administration errors [1-4]. Different studies have used interventions to reduce these errors. These include the development of computerized physician order entry (CPOE), electronic medication administration record (eMAR) systems, automated dispensing cabinets (ADCs), and barcode medication administration (BCMA). As of 2015, more than 98% of hospitals in the United States used ADCs and BCMA, which enable nurses to obtain medicines correctly and reduce errors when medicines are put into the ADC. These technologies have become increasingly prevalent in large hospitals. Literature reviews have shown that this technology has led to a decrease in drug-related errors and has increased the safety of hospital prescription and administration procedures [5-10]. Among these technologies, the automated cabinets used to store and dispense drugs at health care facilities have made it possible to control and monitor drug dispensing. On the basis of literature reviews, the ADC optimizes the inpatient drug administration process, reduces medication errors, and saves time in delivering drugs to and from the pharmacy and waiting for them, making the administration process smooth and safe [11-15]. Although common in the United States, ADC systems are rare in Taiwan because of the investments needed and the considerable organizational changes. However, health professionals are eager for efficient systems adapted to their work settings.

## Methods

### Study Design and Location

The study was conducted at the National Taiwan University Hospital (NTUH), a medical center located in Taipei, Taiwan. An eMAR with a daily unit dose–dispensing system was used where pharmacy staff prepare the drugs required for a 24-hour period. The packages are sorted by medication, according to the physicians’ orders. The medication orders are entered electronically by physicians using prebuilt order sets or individual orders. The orders are sent to the pharmacist automatically via a two-way interface and are then verified by the pharmacist. The nurses use the eMAR to follow the “3 checks and 5 rights” routine and then take out the required medication from the unit dose drug (UDD) cart before administration.

Clinical outcomes, along with patient safety, were assessed, considering a 2-year analytical horizon starting in 2018. The research site was the university hospital, which has 3 locations (East, West, and Children’s Hospital). The East District has 52 wards (13 intensive care units, 39 wards) and 1 pharmacy. The Children’s District has 16 wards, a delivery room, and a newborns room, whereas the West District has 14 wards; the two districts share a medicine storehouse.

To investigate improvements in medication administration by nurses and medication safety using the ADC, we chose one ward in each of the East and West Districts and an intensive care unit in the East District. The A unit in which the ADC was implemented was oncology, which has 35 beds; the B unit has 35 surgical beds; and the C unit was intensive care and has 18 beds. About 80% to 88% of the prescribed drugs were dispensed by the ADC. A longitudinal study was designed using a survey for nurses. The survey was conducted using clinical observations and a questionnaire developed by the nursing information team of the nursing department. The questionnaire was administered in May 2018. An observational study design was used to understand the time differences in drug preparation, delivery, and returns from the inpatient ward by nurses before and after using the ADC. The clinical observations were randomly selected 1 week before and 9 months after initiating the ADC system. The nurses from the 3 units were observed, and the time required for medication preparation and returns was recorded. Medication errors, as recorded by the Medical Incident Events system, were evaluated the year before and after ADC implementation. An anonymous questionnaire was sent to 22 nurses from the intensive care unit in the hospital. These nurses were not included in the final survey. Their comments were considered to see if any amendments to the survey were necessary. The anonymous questionnaire consisted of two parts: (1) the nurses’ demographic characteristics and (2) questions on their perceptions of safety, training, efficiency, timeliness, availability, and accessibility, assessed on a 5-point Likert scale (1=strongly disagree to 5=strongly agree). Reliability was assessed with the Cronbach alpha, which was .92, based on the 19 perception statements. The mean perception score for the 19 items was established; a higher score indicated a higher rate of agreement. The questionnaire was based on Zaidan [16], which includes a total of 21 items covering two aspects: nurse perceptions and satisfaction.

### Ethical Approval

This study was approved by the Research Ethics Committee at the NTUH. The informed consent form was waived (Research Ethics Committee #201807025RINA). All nurses were sent an email explaining the purpose of the study and that they were not obliged to participate. No formal consent form was used, but a returned questionnaire was considered implied consent to participate.

### Data Collection

The following outcomes were considered in the analysis: the time discrepancy in drug preparation, delivery, drug returns from the inpatient, number of ADEs, and the questionnaire results.

The clinical observations conducted included the assessment and time calculations during the nurses’ medication preparation procedures before and after ADC implementation; the measurements were recorded for a total of 6 days (Monday to Saturday). All observers were given instructions before data collection. According to the chosen time for each unit, the observer conducted observations of 3 nurses each day. The medication preparation time was measured as 1 patient for each nurse during the medication preparation process. Drug preparation time started when the eMAR was opened by the nurse and ended when the nurse completed the patient’s medication preparation. After ADC implementation, the starting point of the drug preparation time was when the computer of the ADC was opened. The endpoint was when the nurse completed the medication preparation for the patient, including the medication retrieval process from all necessary retrieval locations, as well as the time spent on the whole process. Medication return was the intact drug package when the patient did not need to use it (eg, pro re nata drugs, that is, medication that is taken as needed). The nurse needed to calculate the number of medications and fill out the drug withdrawal form. The starting time was from the moment when the remaining medicines were taken from the trolley until the quantities of all medicines were filled; this was recorded as the total return time.

The medication administration information was collected from the eMAR database to calculate the delivery time of the medication or first-time use. The starting point was when the physician completed the order, and the endpoint was when the drug was delivered to the unit by the delivery staff. The delivery staff used a mobile phone to scan the barcode of the unit to record the delivery time. After ADC implementation, the time recorded was after the nurse received the physician’s order and started selecting medications from the ADC.

Data concerning medication administration came from the NTUH information system. The error rate was calculated as the number of errors divided by the total opportunity for errors (sum of all doses ordered) multiplied by 10,000. Data concerning an ADE were collected from the adverse event system, which stored the details of each event notification, including the date, place, type of occurrence, drug involved, phase of the process, classification, and type of resulting harm. Events that occurred in the unit 1 year before and after the ADC was implemented were analyzed.

The total number of questionnaires returned was 76, and the return rate was 100%. Unit A was an oncology ward with 16 nurses, unit B a surgical ward with 20 nurses, and unit C an intensive care ward with 40 nurses.

### Data Analysis

Data from the survey were directly exported to SPSS, version 22 (IBM Corp). The data were analyzed using descriptive and inferential statistics, including frequency and percentage, a paired *t* test, and a correlation analysis. A normality test was carried out on the perception score. The significance level was set at an alpha of .05. For open-ended questions, a content analysis was performed. Words and phrases in the open-ended responses were analyzed by team members and then compared.

## Results

### Medication Preparation and Medication Return Time

The time taken to prepare patient medications was recorded for the 3 inpatient wards before and after ADC implementation. The results are shown in Table 1. The medication preparation times of the 3 units for the mean medication preparation time for each patient increased. A paired *t* test showed that all 3 units had a *P* value of <.005. Only 1 unit had an increased drug return time, although the paired *t* test had a *P* value of >.10. Unit A was a surgical ward; most of the patients were there before or after surgery. Although the characteristics of the patients did not change, severity may be different. The drug coverage rate of the ADC was 80%, and there were some medicines that had to be taken out of the medicine cart, which may cause a difference in the return time.

**Table 1:** Comparison of drug preparation and return times.

Item and unit		ADC^a^ implementation		Paired *t* test	*P* value
		Before, mean (SD)	After, mean (SD)		
**Preparation time (min)**					
	A	1.67 (1.37)	4.00 (2.52)	−3.25	.01
	B	0.39 (0.61)	2.11 (1.08)	−5.36	<.001
	C	1.22 (0.94)	2.39 (0.92)	−3.48	<.001
**Return time (min)**					
	A	1.13 (0.52)	1.07 (1.79)	0.14	.89
	B	0.13 (0.52)	0.47 (0.52)	−1.58	.14
	C	1.27 (1.90)	0.47 (1.37)	1.29	.22

^a^ADC: automated dispensing cabinet.

### Urgent Medication Delivery Time

Before ADC implementation, the mean waiting time for urgent medications to be delivered from the pharmacy to the unit was between 10 and 15 minutes. After the ADC was implemented, the most urgent medications were included in the ADC. These were retrieved in a timely manner without waiting for drug delivery. The only waiting time pertained to information transmission from the hospital information system to the ADC, which usually occurred within 3 minutes.

### Medication Error

During the study period, a total of 20 ADEs were reported in the 3 units (Table 2). A total of 9 (45%) adverse events occurred during the drug administration phase and 11 (55%) events during the drug-dispensing phase. Although a decrease in the mean number of events reported was observed between the pre-ADC (12 events/year) and post-ADC (8 events/year) system implementation periods, this difference was not significant.

**Table 2:** Medication error.

Unit	Drug administration phase		Drug-dispensing phase		*P* value
	Before ADC^a^, n	After ADC, n	Before ADC, n	After ADC, n	
A	2	3	6	1	.71
B	2	1	1	3	.78
C	1	0	0	0	.34
Total	5	4	7	4	.77

^a^ADC: automated dispensing cabinet.

### Questionnaire

Of the 76 nurses, 39.5% (n=30) were aged 21 to 30 years, and 48.6% (n=37) had 1 to 5 years of experience. Regarding education level, 92.1% (n=70) had a bachelor’s degree, and 36.8% (n=28) were ranked as N3 nurses based on the clinical ladder system.

The results of the statistical analysis of the questionnaire are shown in Table 3. The overall mean score was 3.90. Among the perceptive aspects concerning the use of ADC, the highest ratings were “I now spend less time waiting for medications that come from the pharmacy than before the ADC was implemented” (score=4.24). The item with the lowest score was “I have to wait in line to get my patient medications” (score=3.32). With regard to accessibility, the item with the highest score was “I am able to select the best available ordered medication” (score=4.22). The item with the lowest score was “I am able to get all of my medications in one place” (score=3.68). The item that received the highest number of complaints in the open-ended questions was “I hope the pharmacist verifies medications faster,” which was raised by 9 (17.6%) participants. A total of 6 (11.7%) nurses mentioned that “The ADC systems and the hospital information system takes too much time to connect.”

**Table 3:** Nurse performance questionnaire results.

Item		Score, mean (SD)
**Nurses’ perceptions**		3.89 (0.77)
	The medication delivery system allows me to do my job more safely.	4.12 (0.65)
	The amount of time between when a written order is sent to the pharmacy and when it is available from the ADC^a^ system is acceptable.	3.57 (0.98)
	I am able to administer meds more efficiently (on time, right dose, etc) with the ADC system.	3.89 (0.72)
	All drawer types assure safe access and removal of medications.	3.93 (0.68)
	There are rarely discrepancies when doing narcotic counts.	4.09 (0.59)
	I now spend less time waiting for medications that come from the pharmacy than before the ADC was implemented.	4.24 (0.73)
	I can confidently use the system after minimal training.	4.05 (0.63)
	The training materials provided were informative and adequate.	4.09 (0.64)
	I have to wait in line to get my patients’ medications.	3.32 (1.24)
	The pharmacist can answer questions and/or solve the ADC system’s problems.	3.68 (0.89)
	The number of phone calls to the pharmacy for requests is acceptable.	3.84 (0.67)
**Accessibility**		3.90 (0.77)
	I have access to all the medications I need.	3.71 (0.89)
	I am able to get all my medications in one place.	3.68 (0.85)
	It is easy to obtain medications during an emergency.	3.87 (0.96)
	Medications are more readily available.	4.14 (0.67)
	The system would work better if more meds were in the ADC system.	4.00 (0.71)
	I am able to select the best available ordered medications.	4.22 (0.53)
	The physical layout of the system is user-friendly.	3.71 (0.73)
	Generally, I am satisfied with the ADC.	3.86 (0.79)
Overall mean score		3.90 (0.77)

^a^ADC: automated dispensing cabinet.

## Discussion

### Principal Findings

#### Impact on Medication Preparation and Medication Return Time

This study examined nurses’ attitudes and workflow after the implementation of an ADC system. The majority of nurses were satisfied with the system, but there was a negative impact on workflow relating to access to medications, as demonstrated by our observations. At our study site, before the implementation of the ADC, the UDD cart stored drugs used by patients throughout the day. The nurse took out the patient-specific pillbox from the medication cart every day and performed the 3 checks and 5 rights of confirmation with the patients. After the implementation of the ADC, because the research unit did not have barcode scanning, after taking out the medicine from the ADC, nurses needed to perform the 3 checks and 5 rights and then perform the routine again when the medicine was distributed to the patient unit to prevent medication errors. Therefore, the preparation time after ADC implementation was significantly longer than before implementation. We found that the preparation time observed in our study was higher than that of previous studies. For example, Franklin et al [17] reported that after implementing a closed-loop ADC system consisting of BCMA, eMAR, and CPOE, the average time per round of dosing was reduced by approximately 10 minutes. Our study did not use BCMA, so nurses needed to perform the 3 checks and 5 rights twice, which may have led to an increase in preparation time.

The ADC systems included 80% to 90% of the medications commonly used in the units, which were retrieved only when needed. Therefore, in most cases, there was no need for medication returns. However, the B unit showed an increased medication return time. After reinspection, we found that a total usage of 1157 pills per 11 types of medications were recorded by the B unit during the study period; among these, 176 (15.2%) pills per 40 (25%) types of medications were not stored in the ADCs, which possibly caused the time increase in medication returns. A descriptive study analysis by Deliberal et al [18] revealed that after the implementation of ADCs, the mean percentage of returned medications decreased from 27% to 4% in the first year and to 4.5% in the second year. Despite differences in scope, the above studies indirectly reflect a possible relationship between the implementation of ADCs and a decrease in the time of returned medications, since medication consumption was reduced after implementation.

#### Rate of Medication Error

**Figure 1:**
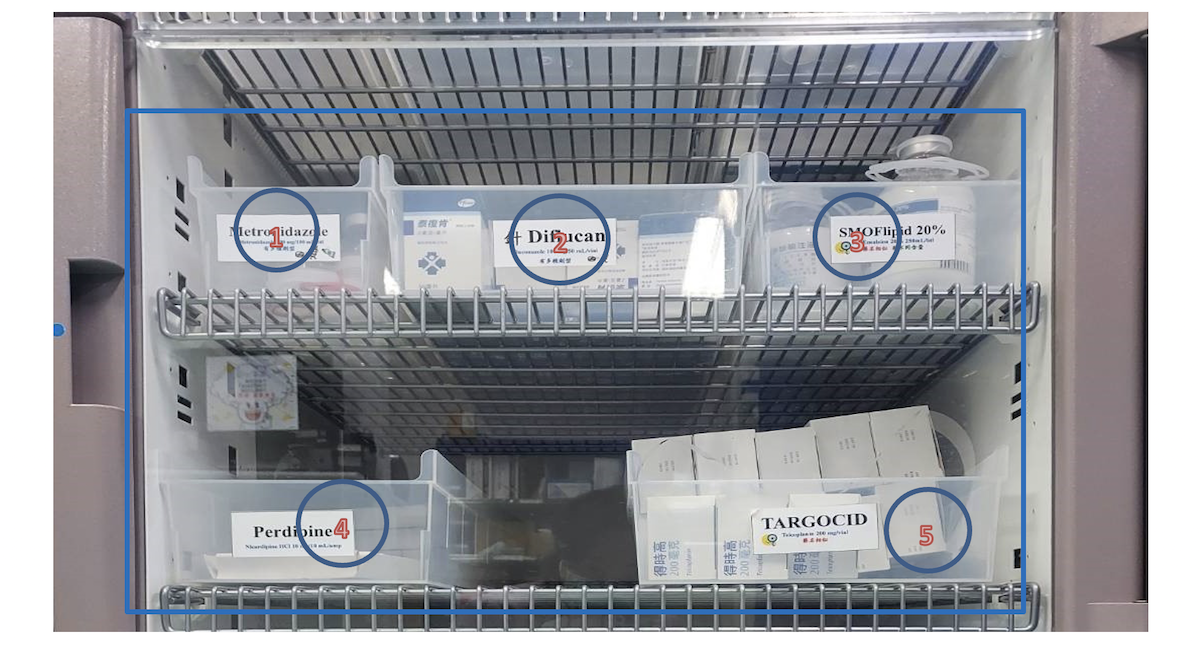
Five medicines are stored in one cabinet (as shown in the square). There is no special device to remind the staff of the location of the medicine. Only by checking the medicine name can they identify that the medicine is correct.

In terms of the medication error rate, only 1 unit showed an increase in the drug-dispensing phase. After analyzing the 4 medication errors in the drug-dispensing phase, it was found that the errors related to the ADC were classified as “dose error and drug error.” There are 2 to 4 kinds of bottled medicines (eg, antibiotics) in the same cabinet. When the medicine cabinet is opened, at least 2 or more drugs must be identified (as shown in Figure 1). The drug will have both the generic name and the brand name, and this may cause the nurse to misidentify the drug when removing the medication. On the ADC screen, the doctor’s orders would read “2 bottles per day” at the top and “take out 1 bottle” below, which may also cause the nurse to administer the wrong dosage if the top instructions go unnoticed. Oldland et al [19] found that the medication error rate when using the UDD alone was 0.157%. After ADC implementation, the comparative overall incidence of error was 0.135%. Subsequent changes in product labeling and more staff training in the use of barcode systems were associated with a decrease in the rate of medication error to 0.050%. Therefore, it can be assumed that the continuous use of barcodes can effectively minimize medication errors.

#### Questionnaire Results

The questionnaire results indicated that the majority of nurses agreed that they could do their job more safely using the ADC system and that it made their job easier. Of the nurses surveyed, 82.9% (n=63) agreed that the drawer types assured safe access and removal of medications. These can provide a higher level of security by allowing access to only one preselected medication at a time. Overall, nursing staff were satisfied with the use of the ADC technology and believed it facilitated their work, helped provide safe patient care, and reduced medication incidents. They could use the system confidently after minimal training, but waiting in line was a major difficulty frequently associated with ADC use. According to the Institute for Safe Medication Practices ADC survey [20], almost one-third of frontline nurses reported always or frequently lining up to access the ADC. Another cross-sectional study also pointed out that 63% of nurses mentioned waiting in line to get patient medications [15].

### Limitations

In this study, only 3 wards from a single medical center were used to explore the time differences before and after ADC implementation; hence, the implications of the research results are limited. The study timeline of the ADC system was about 1 year; therefore, the ADC system can be amended and deficiencies corrected to improve the system in the future. This should improve the system’s efficiency.

### Conclusions

This study explored nursing staff’s perceptions of and satisfaction with an automatic dispensing system in specialized hospitals. The nurses were generally satisfied with the ADCs over the 9 months following complete system implementation and integration. The ADC offered benefits in terms of pharmaceutical stock management [21,22]; however, this came at the cost of increased nursing time. Previously, controlled drugs were stored in lockable drawers. Resupply was performed twice a week and was generated by the nurse. After ADC implementation, the medicine began to be placed in the care unit’s the ADC, resulting in a centralized and closed stock. Resupply, which was automatically generated by the hospital information system, began to be performed once daily and was monitored by the pharmacy team to ensure organization according to the record of each product in the dispensing system. Because this research institution has no configuration to use barcodes, the nursing staff could not use barcode scanning for secondary confirmation when administering drugs and had to manually confirm that the medication name matched the eMAR. Therefore, to reduce the chance of medication ADEs, one should consider the medication packaging, appearance, name, dose, dosage form, and frequency of use when placing medications in the cabinets and stagger the drugs as much as possible or place brightly colored warnings and reminders to reduce nursing staff errors when retrieving medications [16]. Nurses were generally supportive of the benefits of the ADC system to their patients, despite hindrances to their workflows. This study’s findings are indicative of the acceptance of ADCs by nurses.

## Abbreviations

ADC: automated dispensing cabinet
ADE: adverse drug event
BCMA: barcode medication administration
CPOE: computerized physician order entry
eMAR: electronic medication administration record
NTUH: National Taiwan University Hospital
UDD: unit dose drug
